# Antiocclusion Visual Tracking Algorithm Combining Fully Convolutional Siamese Network and Correlation Filtering

**DOI:** 10.1155/2022/8051876

**Published:** 2022-08-09

**Authors:** Xiaomiao Tao, Kaijun Wu, Yongshun Wang, Panfeng Li, Tao Huang, Chenshuai Bai

**Affiliations:** School of Electronic and Information Engineering, Lanzhou Jiaotong University, Lanzhou 730070, China

## Abstract

Machine learning only uses single-channel grayscale features to model the target, and the filter solution process is relatively simple. When the target has a large change relative to the initial frame, the tracking effect is poor. When there is the same kind of target interference in the target search area, the tracking results will be poor. The tracking algorithm based on the fully convolutional Siamese network can solve these problems. By learning the similarity measurement function, the similarity between the template and the target search area is evaluated, and the target area is found according to the similarity. It adopts offline pre-training and does not update online for tracking, which has a faster tracking speed. According to this study, (1) considering the accuracy and speed, the target tracking algorithm based on correlation filtering performs well. A sample adaptive update model is introduced to eliminate unreliable samples, which effectively enhances the reliability of training samples. With simultaneous changes in illumination and scale, fast motion and in-plane rotation IPR can still be maintained. (2) Determined by calculating the Hessian matrix, in the Struck function, Bike3 parameter adjustment can achieve fast tracking, and Boat5 ensures that the system stability is maintained in the presence of interference factors. The position of the highest scoring point in the fine similarity score map of the same size as the search image is obtained by bicubic interpolation as the target position. (3) The parallax discontinuity caused by the object boundary cannot be directly processed as a smooth continuous parallax. The MeanShift vector obtained by calculating the target template feature and the feature to be searched can increase the accuracy by 53.1%, reduce the robustness by 31.8%, and reduce the error by 28.6% in the SiamVGG algorithm.

## 1. Introduction

The tracking algorithm based on the fully convolutional Siamese network (SiameseFC) uses the Siamese network structure to build a tracking framework, which transforms the tracking problem into a similarity measurement problem between sample images and target search regions. The algorithm includes the target sample image, the target search area image, and the CNN network. According to the similarity measurement function, the output features of the upper half and the output features of the lower half are convolved to obtain the similarity, and then the maximum similarity is taken out. Position the maximum similarity and map it back to the original image, and finally use it as the tracking prediction result. By learning the similarity measurement function, the similarity between the template and the target search area is evaluated, and then the target area is found according to the similarity [1–3]. The method of offline pretraining and no online update is used for tracking. Although it has a faster tracking speed, since the target template is not updated online, when the target changes greatly from the initial frame, the tracking effect is poor. The problem of solving the filter is transferred from the time domain to the frequency domain by using the cyclic structure and the Fourier transform. FCNT obtains the filter by multiplying the counterpoints in the frequency domain. When the target search area has the same interference of the target, the tracking result will be poor. Only single-channel grayscale features are used to model the target [4–7]. Although the filter solution process is relatively simple, the tracking effect is poor. It is mainly composed of a recurrent neural network. The algorithm has abstraction for feature extraction module selection, such as minimum output square error and traditional pixel features used by the MOSSE filter. In the field of single-target tracking, the Siamese network framework takes a target template patch *z* (Template patch) and a search area block *x* (Search patch) as input, where *z* represents the target object, and *x* is the larger search area in subsequent video frames. Similar feature maps obtain the classification branch result Cls through CNN and determine the categories of different pixel positions. The KCF algorithm for multichannel HOG features is proposed, and the tracking effect is significantly improved, but the algorithm uses low-dimensional data to represent high-dimensional features when processing multichannel sample features, and the feature information will be lost. The regression branch result Reg is obtained, and the precise position of the tracking target is determined. The tracking algorithm implements the tracking network through classification tasks and regression tasks in the training phase. It is worth noting that the size of *z* is larger than that of the target template block *z* extracted by the Siamese network tracking algorithm. The generative model pays more attention to the description of the target, and the discriminative model pays attention to the classification of the target and the background. The generative modeling is time-consuming and fails to consider the background information. The tracking algorithm based on discriminant correlation filtering performs template matching and background discrimination at the same time. After obtaining the tracking model, the feature responses are obtained [8, 9]. In the visual system, geometric features are the main mechanism for humans to recognize or track objects. When the target is deformed, the depth image features can be used as effective information to assist the visual tracking task, which brings new research directions to visual tracking. The traditional discriminative correlation filtering tracking algorithm solves the regression through the samples generated by the closed-form solution loop in the Fourier domain. Deep learning-based discriminative correlation filtering methods use stochastic gradient descent or conjugate gradient methods to avoid boundary effects. A multicue pedestrian detector and an online detector are jointly used to learn individual object models, incorporating visible light and depth data in the same decision framework. Using RGBD features to build a more stable and more discriminative model, it can effectively identify the target area from the background. When visible light changes in illumination and occlusion, the robustness of the model decreases. The essence of image formation is actually a process of projection [10–13]. It can deal with the change of illumination in visual tracking, and the three-dimensional information of the object is lost in the process. How to restore the three-dimensional information has become the main content of binocular stereo vision research. Based on RGBD data for visual tracking task, a special occlusion template set is designed to supplement the existing dictionary to deal with different occlusion situations. Finally, a depth-based occlusion detection strategy is proposed to determine the template update time. The two-dimensional appearance model and three-dimensional distribution model of the target are simultaneously constructed using visible light and depth images, and the visual tracking task is divided into three parts: detection, learning, and segmentation. The detection and segmentation part uses the above two models to locate the target, and the learning part is used to estimate detections, segmentation errors, and update the target model. The eyes send the collected two-dimensional images to the brain for calculation and processing, and finally form a three-dimensional image of the objective object. The binocular parallax is based on this principle, and the binocular camera is used to obtain two images, one is called the reference image, and the other is called the target map, and the disparity is calculated based on the position difference between the corresponding points in the reference map and the target map. This binocular camera is composed of two monocular cameras with the same parameters on the same horizontal line, and it is used to shoot objects in the real world. Taking multiple photos from different perspectives ensure that the effects of large differences between two photos taken in the same scene due to differences in camera performance and distortion are eliminated. In the test sequence, the result of camera calibration will have a significant impact on the accuracy of subsequent stereo matching, which is an indispensable step in the binocular stereo vision system. In the process of image acquisition, due to the influence of camera distortion, shooting angle, and light and other factors, the collected image may appear partially distorted. On the corrected image pair, after the disparity map of the two images is output in the previous step, the depth information can be determined by combining the internal and external parameters of the camera and the geometric relationship, so as to obtain its real coordinates in the real space, and finally realize the 2D image to 3D scene output update target. The advantage of particle filter is that it has better modeling ability in nonlinear environment, so it performs well in the field of target tracking. The discriminant model directly obtains the decision function from the limited data and directly learns the conditional probability distribution from the perspective of probability. The sparsity of target candidates is achieved using the least squares method. By splicing visible light and thermal infrared features together, both thermal infrared features and visible light features may exist as noise, thermal infrared features in thermal crossover or visible light features in dark conditions, etc. From a machine learning perspective, discriminative tracking is essentially a regression or classification problem [14, 15]. The background information of the image is introduced into the discriminative model. Modeling the object appearance usually uses sparse or low-rank theory to learn the representation coefficients of the features, taking into account the background noise. Some algorithms will use the target and background of recent frames to update the target model at regular intervals during the tracking process. Compared with the generative model, the discriminant has higher robustness to the interference of external factors and its own deformation. Therefore, the input target features should be comprehensively analyzed to greatly improve the robustness of target modeling. The previous algorithms for constructing the target appearance representation usually directly input the target original features without decomposing them to obtain the target appearance model.

## 2. Siamese Network Class Tracking Algorithm

### 2.1. The Principle of Binocular Stereo Vision

**Figure 1:**
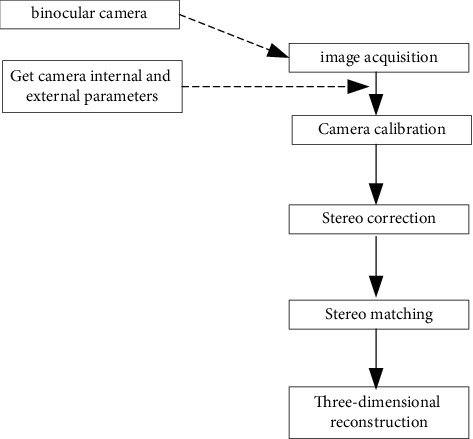
Binocular stereo vision system.

Epipolar constraint is one of the most critical constraints in stereo matching. The line segment connecting the optical center of the camera is called the baseline. Abstract function represents the similarity score. But in the case of binocular cameras, a point in the real scene will form two different images on the image plane of the left camera and the image plane of the right camera, respectively. According to the position offset between the two images, plus the camera model and the geometric relationship between them, the depth information of the point can be calculated as shown in Figure 1.

### 2.2. RGBT-Based Visual Tracking Technology

**Figure 2:**
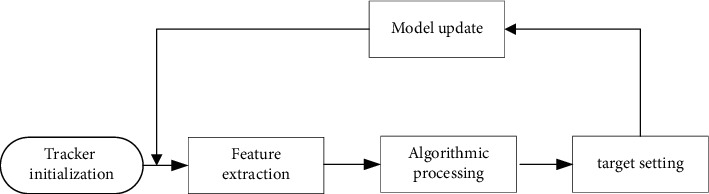
Visual tracking algorithm framework.

Depth information can provide valuable features to assist trackers in predicting target locations when dealing with visual tracking tasks. It can not only increase the training speed but also solve the gradient vanishing problem of the sigmoid function. The depth sensor is limited by a limited range, and in practical applications, the RGBD visual tracking algorithm has many limitations. The depth sensor only collects the distance between the scene and the image collector, and it cannot correctly distinguish targets with the same distance, as shown in Figure 2. In recent years, visual tracking based on visible light modality has developed rapidly, and a large number of labeled datasets have been proposed for training models. The visual tracking technology based on RGBT was developed relatively late, and a dataset with ground-truth registration of visible light and thermal infrared was constructed. Dropout is used to alleviate over fitting. The training set is isolated from the test set of target tracking, the test results of the algorithm are more credible, and the ILSVRC dataset is established for the video target detection problem. The background between different frames is linearly correlated, and the moving target appears relatively sparse, so the model of the target appearance is usually based on the theory of sparse representation. Sparse representation to multimodal data fusion is applied, and visible light and thermal infrared reference templates are updated in a jointly optimized way for visual tracking tasks.

## 3. Algorithm Model

### 3.1. SiamVGG Algorithm [16–20]

Fully convolutional Siamese network

(1) $$\begin{array}{c} \text{dist}\left(X,Y\right)=\sqrt{\sum_{i=1}^{n}{\left(x_{i}-y_{i}\right)}^{2}}. \end{array}$$

Tracking algorithm

(2) $$\begin{array}{c} \text{sim}\left(X,Y\right)=\cos\;\theta =\frac{x•y}{\left\|x\right\|•\left\|y\right\|}. \end{array}$$

Sample image

(3) $$\begin{array}{c} \text{output}=\sum_{i=1}^{m}\sum_{j=1}^{n}I_{i,j}\times K_{i,j}+b. \end{array}$$

Target search area

(4) $$\begin{array}{c} o=\frac{i+2p-k}{s}+1. \end{array}$$

(5) $$\begin{array}{c} \mu_{B}\leftarrow \frac{1}{m}\sum_{i=1}^{m}x_{i}. \end{array}$$

Similarity

(6) $$\begin{array}{c} \sigma_{B}^{2}\leftarrow \frac{1}{m}\sum_{i=1}^{m}{\left(x_{i}-\mu_{B}\right)}^{2}. \end{array}$$

(7) $$\begin{array}{c} x_{i}\leftarrow \frac{x_{i}-\mu_{B}}{\sqrt{\sigma_{B}^{2}+\varepsilon }}. \end{array}$$

Predicted location

(8) $$\begin{array}{c} y_{i}\leftarrow \gamma x_{i}+\beta . \end{array}$$

(9) $$\begin{array}{c} y=\frac{1}{1+e^{-x}}. \end{array}$$

Offline pretraining

(10) $$\begin{array}{c} y=\max\left(0,x\right). \end{array}$$

(11) $$\begin{array}{c} H\left(x\right)=F\left(x\right)+x. \end{array}$$

Loop structure

(12) $$\begin{array}{c} f\left(z,x\right)=\varphi \left(z\right)*\varphi \left(x\right)+b. \end{array}$$

(13) $$\begin{array}{c} l\left(y,v\right)=\log\left(1+\exp\left(-yv\right)\right). \end{array}$$

### 3.2. SA-Siam Algorithm [21–23]

(14) $$\begin{array}{c} L\left(y,v\right)=\frac{1}{D}\sum_{u\in D}\left(l\left(y\left(u\right),v\left(u\right)\right)\right). \end{array}$$

MOSSE filter

(15) $$\begin{array}{c} y\left(u\right)=\left\|u-c\right\|\leq R. \end{array}$$

Multichannel HOG features

(16) $$\begin{array}{c} V_{\text{CLE}}=\sqrt{{\left(x_{A}-x_{G}\right)}^{2}+{\left(y_{A}-y_{G}\right)}^{2}}. \end{array}$$

KCF algorithm

(17) $$\begin{array}{c} IoU=\frac{B_{A}\cap B_{G}}{B_{A}\cup B_{G}}. \end{array}$$

2D appearance model

(18) $$\begin{array}{c} \Phi \left(i\right)=\frac{1}{N_{\text{rep}}}\sum_{i=1}^{N_{\text{rep}}}\Phi \left(i,k\right). \end{array}$$

### 3.3. ECO Algorithm [24–27]

(19) $$\begin{array}{c} \rho_{A}\left(i\right)=\frac{1}{N_{\text{valid}}}\sum_{t=1}^{N_{\text{valid}}}\Phi \left(i\right). \end{array}$$

Return branch result Reg

(20) $$\begin{array}{c} \rho_{R}\left(i\right)=\frac{1}{N}\sum_{k=1}^{N_{rep}}F\left(i,k\right). \end{array}$$

3D distribution model

(21) $$\begin{array}{c} \phi_{N_{s}}=\frac{1}{N_{s}}\sum_{i=1}^{N_{s}}\phi_{N_{c}}. \end{array}$$

Classification task

(22) $$\begin{array}{c} \phi =\frac{1}{N_{hi}-N_{lo}}\sum_{N_{s}=N_{lo}}^{N_{hi}}\phi_{N_{s}}. \end{array}$$

Return task

(23) $$\begin{array}{c} x^{\prime }=\rho \left(x;M\right). \end{array}$$

Visual system

(24) $$\begin{array}{c} M_{i}=F_{ap}\left(\frac{\partial J}{\partial F_{i}}\right). \end{array}$$

Light change

(25) $$\begin{array}{c} L={\sum_{i,j}\left\|Y\left(i,j\right)-W*X_{i,j}\right\|}^{2}+\lambda {\left\|W\right\|}^{2}. \end{array}$$

Closed loop

(26) $$\begin{array}{c} f\left(z,x\right)=\sum_{i,j}\varphi \left(z\right)*\varphi \left(x\right)+bI. \end{array}$$

(27) $$\begin{array}{c} R=\arg_{T}\min{\left\|T*X-Y\right\|}^{2}+\lambda {\left\|T\right\|}^{2}. \end{array}$$

## 4. Simulation Experiment

### 4.1. Generative Models

**Figure 3:**
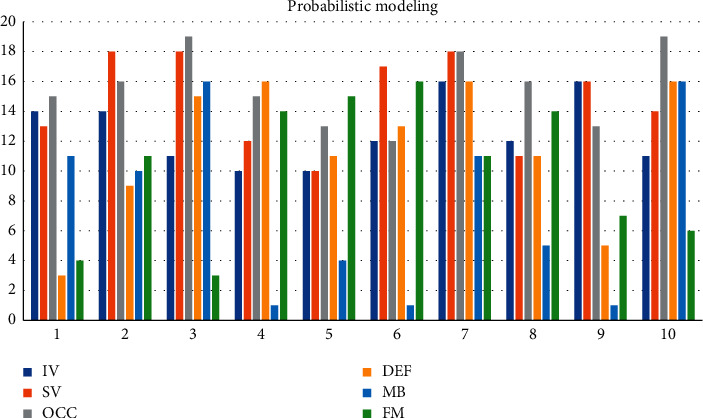
Probabilistic modeling.

**Figure 4:**
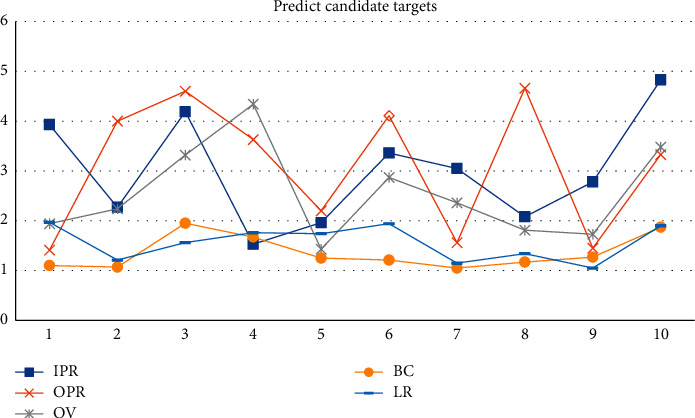
Predicting candidate targets.

In generative models, statistical models are usually generated from a probabilistic perspective, using past information to train a joint probability distribution, and modeling the posterior probability to predict the categories of candidate targets, as shown in Table 1 and Figures 3 and 4. Consider IV = 14, SV = 13, OCC = 15, and DEF = 3 in terms of accuracy and speed. The performance of the target tracking algorithm based on correlation filtering is relatively good. MB = 11, FM = 4, and IPR = 3.93. Most of the current tracking algorithms select the target as the center to cut out a fixed proportion of the area to be searched. The generative model uses the historical frame information to characterize the target and finds the candidate target with the smallest reconstruction error as the new target. OPR = 1.41, OV = 1.94, BC = 1.1, and LR = 1.97. Whether the search locale is set properly has a lot to do with the correct tracking. Depending on the sample size, the central target is cyclically shifted to obtain a set of positive and negative samples. Due to the existence of negative samples, the correlation filter can distinguish the target and the background well. Considering the background information and the diversity of changes in the target's own appearance, the results obtained by applying multiple trackers in the target decision-making layer are used as the final result. The sample adaptive update model is introduced to eliminate unreliable samples and effectively enhance the reliability of training samples. The illumination change IV = 10, the scale change SV = 10, the occlusion OCC = 13, the deformation DEF = 11, and the motion blur MB = 4. Rapid motion FM = 15, in-plane rotation IPR = 1.96, out-of-plane rotation OPR = 2.2, target out-of-view OV = 1.43, background interference BC = 1.25, and low-resolution LR = 1.74.

### 4.2. Kernel Function

**Figure 5:**
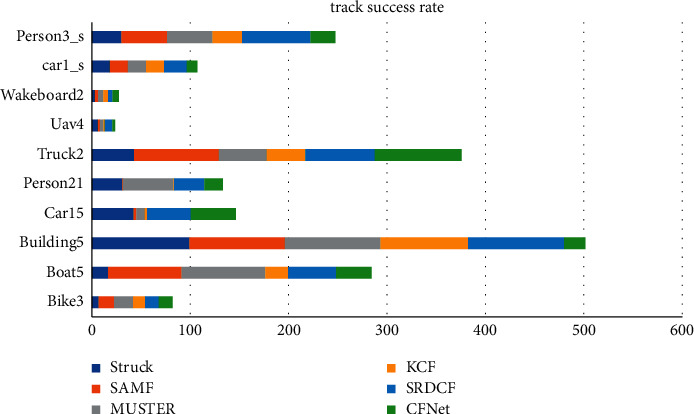
Tracking success rate.

The target image is obtained, the depth feature of the template image is calculated by the feature extraction network, and the search image is obtained during subsequent continuous tracking. Depth features are computed through the same feature extraction network, as shown in Table 2 and Figure 5. The feature extractor uses the VGGNet deep network, and then maps the feature maps of different layers to the continuous confidence map in the spatial domain, and finally the center position of the target is determined by calculating the Hessian matrix. In the Struck function, Bike3 = 6.9, Boat5 = 16.6, and Building5 = 99.3. A fine similarity score map of the same size as the search image is obtained by bicubic interpolation Car15 = 42.4, Person21 = 31.2, Truck2 = 42.9, Uav4 = 6.3, Wakeboard2 = 3.1, car1_s = 18.4, and Person3_s = 30.2 The position of the highest scoring point is the target location. By analyzing and calculating the similarity and correlation of adjacent video frames, the estimation of the target position state is realized. The disadvantage of the C-COT algorithm is that the amount of computation and data is very large when training with the VGGNet deep network, which makes it difficult to meet the real-time requirements of target tracking. The assumptions of the method based on optical flow are two points: one is that the brightness of the target is constant when moving, and the other is that the gap between the adjacent video frames is small.

### 4.3. Parallax Continuity Constraint

**Figure 6:**
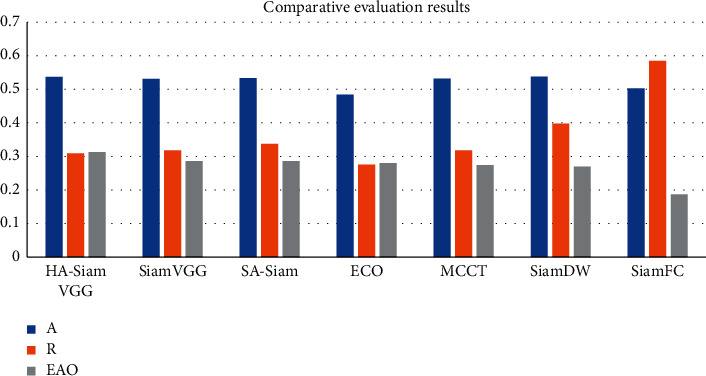
Comparative evaluation results.

Calculate the pixel feature value probability of the target and the frame to be searched to obtain the template and the feature model to be searched. According to the constraint rule that parallax has continuity, the parallax on the surface of this object is considered to be continuous and smooth, as shown in Table 3 and Figure 6. In the HA-SiamVGG calculation template, *A* = 0.537, *R* = 0.309, and EAO = 0.313. The parallax discontinuity caused by the object boundary cannot be directly processed as a smooth continuous parallax, due to the different shooting angles and the influence of the front and rear occlusion of the object. That is, the mapping point of a point in the space on the left and right image planes is unique. The target frame of the area to be searched is iterated continuously along the vector direction closest to the target in the first frame, and the convergence result is finally obtained through continuous iteration to locate the target. The pixel regions generated by the projection of the same object under different shooting angles must have consistent or similar properties. Due to the influence of the camera's photosensitive components, the surrounding environment, and noise, when the pixels in the same space are mapped to the two-dimensional image, the gray value of the pixels will be different. Similarity constraints should be used to make their corresponding matching points have similar properties. Difficult samples in network training are added, data augmentation to solve the spatial location bias of training is used, and the generalization ability of the model is improved.

### 4.4. Siamese Region Candidate Network Tracking Algorithm

**Figure 7:**
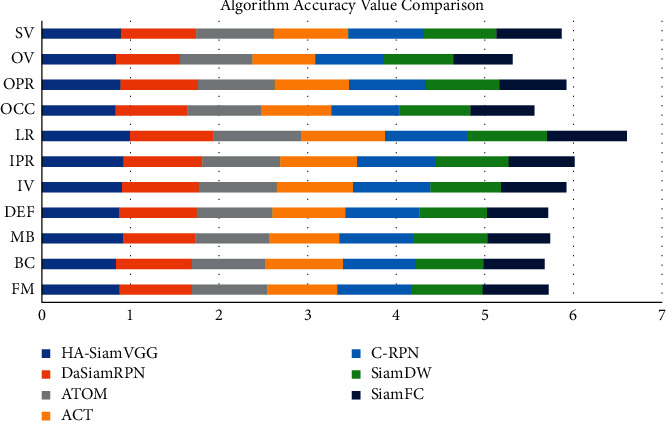
Comparison of algorithm accuracy values.

The confidence of the classified samples is obtained by correlation filtering, and then the target is tracked. The use of signal operations such as fast Fourier transform and dot product greatly improves the real-time performance of target tracking. The depth features are extracted through the same feature extraction network in SiamFC, and the classification branch depth feature map and the regression branch depth feature map are obtained through the RPN network, as shown in Table 4 and Figure 7. Optical flow refers to the use of images to represent the speed of motion, and each pixel is given a speed vector including size and direction. The motion state of the target is judged by the displacement change of the pixels in the adjacent frame images, so as to realize the tracking of the target. The correlation calculation with the template feature is performed to obtain the result feature map of the classification branch and the regression score. It needs to meet the time continuous or the target moves slowly, so the scope of application is small. The MeanShift algorithm is based on the probability density distribution, uses color features to describe the target, and iteratively finds the local optimum along the gradient ascending direction, that is, the position of the target. SiamFC searches based on the scale pyramid method. The calculation of depth features at each scale is time-consuming, and the tracking speed is slow. The introduction of the RPN structure enables SiamRPN to avoid time-consuming multiscale calculations and replace it with bounding box regression, which improves the tracking speed. The algorithm calculates the probability distribution of color features within the target and candidate regions, respectively. The Kalman filter tracking model is used to model the motion as a linear system. The motion state of the target in the current frame depends on the state of the previous frame. The filter is divided into two parts: prediction and observation. Calculate the observed values (speed, acceleration, etc.), and synthesize the observed state and the predicted state to obtain the optimal state estimate. It is not sensitive to the change of the appearance of the target but has higher requirements on the selection of the motion model and the matching of the noise covariance, and the effect becomes worse when the motion speed is fast.

## 5. Conclusion

Traditional image acquisition refers to the process of image acquisition of objects in the real scene, while binocular stereo vision uses a binocular camera to achieve this process. Although machine learning has a faster tracking speed, its shortcomings are also obvious. The disadvantages of machine learning are that it takes a lot of time to make machine programs, and the demand for data is huge. Results may be satisfactory, but a fully automated system requires extensive research and analysis. The backups and servers required to maintain and record the acquired data keep piling up, making it increasingly costly. Since the target template is not updated online, when the target changes greatly from the initial frame, the tracking effect is poor. When there is the same kind of target interference in the target search area, the tracking results will be poor. Only single-channel grayscale features are used to model the target, and the filter solution process is relatively simple. The tracking algorithm based on the fully convolutional Siamese network can solve these problems. By learning the similarity measurement function, the similarity between the template and the target search area is evaluated, and the target area is found according to the similarity. It adopts offline pretraining and does not update online for tracking, which has a faster tracking speed. According to this study: 1. Considering the accuracy and speed, IV = 14, SV = 13, OCC = 15, and DEF = 3. The performance of the target tracking algorithm based on correlation filtering is relatively good. MB = 11, FM = 4, and IPR = 3.93. Most of the current tracking algorithms select the target as the center to cut out a fixed proportion of the area to be searched. The generative model uses the historical frame information to characterize the target, and finds the candidate target with the smallest reconstruction error as the new target. OPR = 1.41, OV = 1.94, BC = 1.1, and LR = 1.97. The sample adaptive update model is introduced to eliminate unreliable samples and effectively enhance the reliability of training samples. The illumination change IV = 10, the scale change SV = 10, the occlusion OCC = 13, the deformation DEF = 11, and the motion blur MB = 4. Fast motion FM = 15, in-plane rotation IPR = 1.96, out-of-plane rotation OPR = 2.2, target out-of-view OV = 1.43, background interference BC = 1.25, and low-resolution LR = 1.74.2 are determined by calculating the Hessian matrix. In the Struck function, Bike3 = 6.9, Boat5 = 16.6, and Building5 = 99.3. A fine similarity score map of the same size as the search image is obtained by bicubic interpolation Car15 = 42.4, Person21 = 31.2, Truck2 = 42.9, Uav4 = 6.3, Wakeboard2 = 3.1, car1_*s* = 18.4, and Person3_*s* = 30.2, and the position of the highest scoring point is the target location. 3. In the HA-SiamVGG calculation template, *A* = 0.537, *R* = 0.309, and EAO = 0.313. The parallax discontinuity caused by the object boundary cannot be directly processed as a smooth continuous parallax, due to the different shooting angles and the influence of the front and rear occlusion of the object. The target template feature is calculated, and the MeanShift vector obtained by the feature is to be searched. In the SiamVGG algorithm, *A* = 0.531, *R* = 0.318, and EAO = 0.286. The visual tracking algorithm is still an active research direction in the field of computer vision. Although detection algorithms have achieved good results, there is still a certain gap in the application in real scenes, and the basic task of target detection is still very challenging. There is great potential and space for improvement.

## Figures and Tables

**Table 1:** Generative models.

IV	SV	OCC	DEF	MB	FM	IPR	OPR	OV	BC	LR
14	13	15	3	11	4	3.93	1.41	1.94	1.1	1.97
14	18	16	9	10	11	2.27	4	2.24	1.07	1.21
11	18	19	15	16	3	4.19	4.6	3.32	1.95	1.56
10	12	15	16	1	14	1.53	3.63	4.34	1.67	1.76
10	10	13	11	4	15	1.96	2.2	1.43	1.25	1.74
12	17	12	13	1	16	3.36	4.11	2.87	1.21	1.94
16	18	18	16	11	11	3.05	1.56	2.36	1.05	1.15
12	11	16	11	5	14	2.08	4.66	1.81	1.17	1.34
16	16	13	5	1	7	2.78	1.45	1.73	1.27	1.05
11	14	19	16	16	6	4.83	3.33	3.48	1.87	1.9

**Table 2:** Tracking success rate.

	Struck	SAMF	MUSTER	KCF	Srdcf	CFNet
Bike3	6.9	15.7	19.4	12.2	13.9	14.1
Boat5	16.6	74.7	85.1	23.2	48.6	36
Building5	99.3	96.9	97.3	89	97.5	21.6
Car15	42.4	3	8.5	2.3	44.4	45.8
Person21	31.2	0.6	51.3	0.6	30.8	18.6
Truck2	42.9	86.1	48.9	39.2	70.5	88.2
Uav4	6.3	1.9	3.8	1.3	7.6	2.8
Wakeboard2	3.1	3.3	5.3	4.9	4.5	6.4
car1_s	18.4	18.4	18.4	18.4	23.2	10.6
Person3_s	30.2	46.5	46.1	30	69.5	25.1

**Table 3:** Comparative evaluation results.

	A	R	EAO
HA-SiamVGG	0.537	0.309	0.313
SiamVGG	0.531	0.318	0.286
SA-Siam	0.533	0.337	0.286
ECO	0.484	0.276	0.28
MCCT	0.532	0.318	0.274
SiamDW	0.538	0.398	0.27
SiamFC	0.503	0.585	0.187

**Table 4:** Comparison of algorithm accuracy values.

	FM	BC	MB	DEF	IV	IPR	LR	OCC	OPR	OV	SV
HA-SiamVGG	0.878	0.836	0.916	0.873	0.905	0.922	0.996	0.833	0.887	0.839	0.892
DaSiamRPN	0.817	0.856	0.816	0.877	0.868	0.889	0.942	0.811	0.877	0.72	0.852
ATOM	0.851	0.827	0.839	0.854	0.881	0.881	0.993	0.832	0.867	0.821	0.876
ACT	0.791	0.881	0.79	0.826	0.86	0.867	0.943	0.799	0.837	0.707	0.841
C-RPN	0.832	0.823	0.831	0.831	0.875	0.89	0.927	0.764	0.865	0.777	0.854
SiamDW	0.808	0.762	0.841	0.765	0.795	0.823	0.902	0.8	0.83	0.78	0.818
SiamFC	0.743	0.69	0.705	0.69	0.736	0.742	0.9	0.722	0.756	0.669	0.735

## Data Availability

The experimental data used to support the findings of this study are available from the corresponding author upon request.
